# Redefining respite for family caregivers: lessons learned from the COVID-19 pandemic

**DOI:** 10.3389/frhs.2025.1611360

**Published:** 2025-07-25

**Authors:** Rebecca L. Utz, Hannah L. Mundinger, Amber Thompson, Gail L. Towsley, Kara B. Dassel, Alex Terrill, Alycia A. Bristol

**Affiliations:** ^1^ Department of Sociology, University of Utah, Salt Lake City, UT, United States; ^2^ College of Nursing, University of Utah, Salt Lake City, UT, United States; ^3^ Gerontology Interdisciplinary Program, University of Utah, Salt Lake City, UT, United States; ^4^ Division of Occupational & Recreational Therapy, College of Health, and Division of Rehabilitation Medicine, School of Medicine, University of Utah, Salt Lake City, UT, United States

**Keywords:** respite, family caregivers, caregiver burden, COVID, long-term supports and systems (LTSS), long-term care (LTC), home-and community-based services

## Abstract

**Introduction:**

Respite care is provided to caregivers through in-home respite providers, drop-off day centers, and institutional or overnight facilities, where the caregiver can take a break or get time-away, while the care-recipient is provided with personal care, companionship, and/or supervision. During the global COVID-19 pandemic (2020+), these types of formal respite services were disrupted, leaving caregivers with little to no access to respite. This study aimed to understand how caregivers accessed and achieved respite when respite services were not readily available, and how their experiences during and following the unprecedented global public health crisis have influenced and informed the way that caregivers achieve the needed and desired respite.

**Methods:**

This study integrates several sources of qualitative and descriptive data collected via electronic surveys and semi-structured interviews with family caregivers and respite providers over the past several years (from 2019 to 2024).

**Results:**

The following themes were identified: (a) disruption and loss of formal respite services resulted in caregiver isolation, as well as acute and protracted stress, (b) personal networks and shared caregiving arrangements provide opportunities for informal respite, (c) respite is associated improved caregiver wellbeing, but caregivers often are hesitant to use respite (d) daily activities and routines can provide a form of respite for caregivers, (e) family caregivers showed resilience and adaptability in the face of COVID-19 challenges, revealing the potential benefit of taking “short breaks” throughout the day to achieve a feeling of respite.

**Discussion and conclusions:**

These qualitative, descriptive insights provide a blueprint for a reimagined definition of caregiver respite, where respite is conceptualized as an outcome or benefit that caregivers seek and can create on their own, rather than only defining respite as a formal service provided to caregivers by outside organizations. In the face of significant workforce shortages that threaten the widespread availability and access to formal respite services, a re-imagined model of respite has the potential to better meet the respite needs and wishes of family caregivers, and maximize the benefit of short-breaks when formal respite services may not be available.

## 1 Introduction

Approximately 53 million Americans, or about one in every five adults, are currently supporting and caring for someone with disability and/or serious, complex illness (1). These *caregivers* are supporting family members, friends, and neighbors by providing personal care, transportation, financial planning, and meal assistance; by delivering and managing complex medical tasks and treatment regimens in the home; as well as by participating in medical decision-making and planning (2). The average family caregiver provides more than 20 h of direct-care per week, with some caregivers reporting round-the-clock or constant need for vigilance and support (1). The economic value of family caregiving in America is estimated at nearly $500 billion annually, an amount that surpasses out-of-pocket and federal spending on long-term care (3). Unfortunately, this largely unpaid workforce often reports significant levels of stress and burden associated with their caregiving role; extant research has established that caregivers may be providing this care at the expense of their own physical, emotional, social, and/or financial health (4).

Recognizing the invaluable, yet personally challenging role that family caregivers fill in society, several policies have defined and prioritized the need to support family caregivers, including the 2022 U.S. National Strategy to Support Caregivers (https://acl.gov/CaregiverStrategy). This document outlines hundreds of federal actions and priorities that can be taken to increase public awareness and support for family caregivers in our society. Among the proposed actions are numerous calls to develop, evaluate, and increase access to specific services and supports for caregivers (5–8). From the perspective of family caregivers, *respite* is among the most desired and requested forms of caregiver support (9–11). Respite is defined as “planned or emergency care provided to a child or adult with a special need in order to provide temporary relief to the family caregiver of that child or adult” (12). In America, there are three primary types of respite care:

1. *In-Home Respite:* scheduled visits from a home health aide or in-home respite provider, allowing caregiver to get-away for a few hours while the care-recipient receives companionship or personal care
2. *Day Programs:* drop-off service where care-recipient receives professional care and supervision in a community-based group setting and caregiver receives extended block of time (i.e., to run errands, to rest, to cover caregivers’ workday)
3. *Institutional Respite:* 24-h residential care at hospital, nursing home, or assisted living facility, where caregiver gets extended break overnight or weekend (i.e., for vacation) while care-recipient temporarily resides in a supportive and safe environment

Respite care provides the greatest benefit to caregivers if used regularly as preventative service (11, 13, 14). That said, many caregivers are hesitant to use formal respite care because they feel guilty asking for help or admitting they need a break; they may doubt the respite care workers’ ability to deliver competent care; or they feel burdened by required paperwork to determine eligibility (15–18).

In America, few health insurances cover respite care, often requiring families to pay out-of-pocket for services (19). Long waitlists for many of the affordable or subsidized respite services, compounded by shortages in the direct-care workforce who would serve as respite providers, have further reduced access to respite services for family caregivers. Accordingly, some have called for new research and innovations that can maximize the benefit of respite services for caregivers, given that demand likely outpaces supply and given that caregivers are often hesitant to fully engage with formal respite care (20–25).

The emergence of COVID-19 in Spring 2020, followed by a multi-year continuation of an unprecedented global pandemic that included stay-at-home directives, physical distancing mandates, and the shut-down of many business (including respite care providers and other forms of long term services and supports, LTSS) provided a unique opportunity from which to explore and better understand how caregivers can achieve respite, especially in an environment where access to formal services may be limited, either by a pandemic or by limited availability of services. A number of research studies have documented the myriad challenges faced by family caregivers during the COVID-19 pandemic, with consensus that COVID-19 made the challenging caregiving role even more difficult, with the addition of new, significant stressors that isolated family caregivers from needed supports and services (26, 27). The goal of this paper is to not simply describe how caregivers coped under the stresses of the public health crisis; instead, this paper describes the experiences and reflections of both caregivers and respite providers during and following this unprecedented time of the COVID-19 public health challenge. Disruptions in LTSS were often met with creativity and resilience, which have led to insights and lessons learned about how respite – as both a formal component of the LTSS industry and as an outcome or feeling that caregivers are seeking – is being redefined and reimagined. The context and data for this study is focused on America, but the description of respite as coming from both formal services and informal shared care arrangements and the emergence of caregivers using daily routines, intentional short breaks, and self-care practices to offset the daily stresses of caregiving is likely applicable to all types of caregivers in all types of circumstances and cultural settings.

## 2 Method

This study uses a sequential mixed-methods research design to build a descriptive, data-informed case-study. It integrates three separate data sources collected over the course of a five-year period (2019–2024). All study procedures for each of the three separate data sources were approved by the University of Utah Institutional Review Board, and have been described elsewhere.

The first data source included an electronic-survey of family caregivers fielded in early summer 2020 (*n* = 82) followed by semi-structured telephone interviews conducted with a subsample of the original sample in the fall of 2020 (*n* = 28) (28, 29). This study was focused exclusively on how caregivers were coping with the challenges of the early-stages of the COVID-19 pandemic; the survey included 47 fixed-choice and closed-ended questions, while interviews ranged in length from 20 to 90 min. The sample included those caring for parents/parents-in-law (45.1%), spouses/partners (26.8%), children (18.3%), and other family members such as siblings and grandparents (9.8%). Over half of participants (53.2%) provided more than 20 h of care per week and the majority (73.4%) lived with the care-recipient. Most care recipients were older (63.8% were over the age of 60); they had neurological conditions (40.6%), disability, and other chronic conditions such as diabetes, heart disease, or cancer (24.8%).

The second and third source of data both came from the Time for Living and Caring (TLC) study, an NIA-funded clinical trial where dementia caregivers used a “virtual coach” (app) to record their weekly respite time and respite time-use satisfaction (NIA: R01-AG061946; Clinical Trials: NCT-03689179) (30). The TLC study ran from September 2019 to May 2024, providing a novel opportunity to observe dementia caregivers, across a five-year period capturing the start of the COVID pandemic and extending to several years later where we have had to learn to live with COVID. Given the serendipitous timing of this study, the TLC study team developed and added a supplemental set of survey questions (7 fixed-choice and 4 open-ended) that focused specifically on how caregivers were affected by the COVID pandemic. The TLC sample includes 163 dementia caregivers who were all using respite or who wanted to become more consistent in their use of formal respite services. They were, on average, 61.7 years of age (standard deviation 13.0, min 20, max 92). They were primarily spouse or partner (68.1%) or adult child (24.1%) to care-recipient. Most were female (78.9%), White (82.5%), non-Hispanic (90.4%), married or living with partner (83.7%), had a college education (89.7%), and incomes greater than $50,000 annually (73.6%).

The TLC study included data collection from respite service providers, providing the third source of data used to build this case-study. Respite providers were primarily asked about the TLC app and how they might utilize this product with their clients, but like the TLC caregiver survey, the provider survey included a few explicit questions asking them to reflect on how COVID affected their clients’ use of respite, and how providers had to adapt to the changing landscape of caregiving and respite-use during and following the COVID pandemic. These data were collected in 2023 and 2024, primarily via an electronic survey. The survey sample includes 58 formal respite services providers who had been providing respite for an average of 11 years (6.5 median). About two-thirds (68%) provided in-home respite; one-third (31%) offered adult day services, and 6% offered overnight or institutional respite. The TLC study also included a series of focus groups and public forums, where the TLC study results (overall) were shared with and discussed with respite providers (i.e., a webinar with Lifespan Respite Providers; presentation at a national conference of respite providers). These interactive sessions provided additional qualitative data for this case study, as the participants in these session often wanted to discuss how they were serving caregivers in light of the changes associated with the COVID pandemic. No formal demographics were collected for these participants. All providers were recruited with the help of the ARCH National Respite Resource Center, and represent both the aging-services and disability-services sectors.

Results are based on descriptive analysis. Survey data are described using frequencies and means. Qualitative data were analyzed and are reported descriptively. All members of the research team, independently and then jointly, reviewed the verbatim interview transcripts and open-ended survey responses using an inductive content analysis technique, which produced five descriptive themes that help organize the data and presentation of results: (1) loss of formal respite care, (2) importance of informal respite (social support), (3) daily routines as a form of respite, (4) challenges of caregiving without access to respite, (5) creativity, resilience, and adaptability. Our qualitative descriptive approach allows for participants’ language to create a data-near and comprehensive summary of the topic, with little need for investigator inference or predetermined theoretical framework (31). The mixed methods results from the three samples are integrated together, rather than being described separately, to provide a single narrative about the use, value, preferences, and needs for respite. Where relevant, the data source is noted for each narrative response or set of data described.

## 3 Results

This case study integrates data across time and samples to describe how caregivers use respite and how they adapted when respite was not available.

### 3.1 Loss of formal respite care

Throughout the COVID-19 pandemic, especially during the very early stages where stay-at-home directives dominated the public health messaging and prior to any vaccines that allowed for some risk protection for the general population, family caregivers spoke frequently about not having access to formal respite care during the pandemic, and how stressful this was for them. A husband caring for his wife said, “*Going weeks without, or very little, respite required much self-control and mental preparedness to get through the day/week (or) month*”. A caregiver to a child with autism was desperate for the return of formal services: “*My mind is grasping for some kind of end date … Literally, every night in my prayers I'm like please, let things calm down. …. I would kill for respite care right now*”.

**Figure 1:**
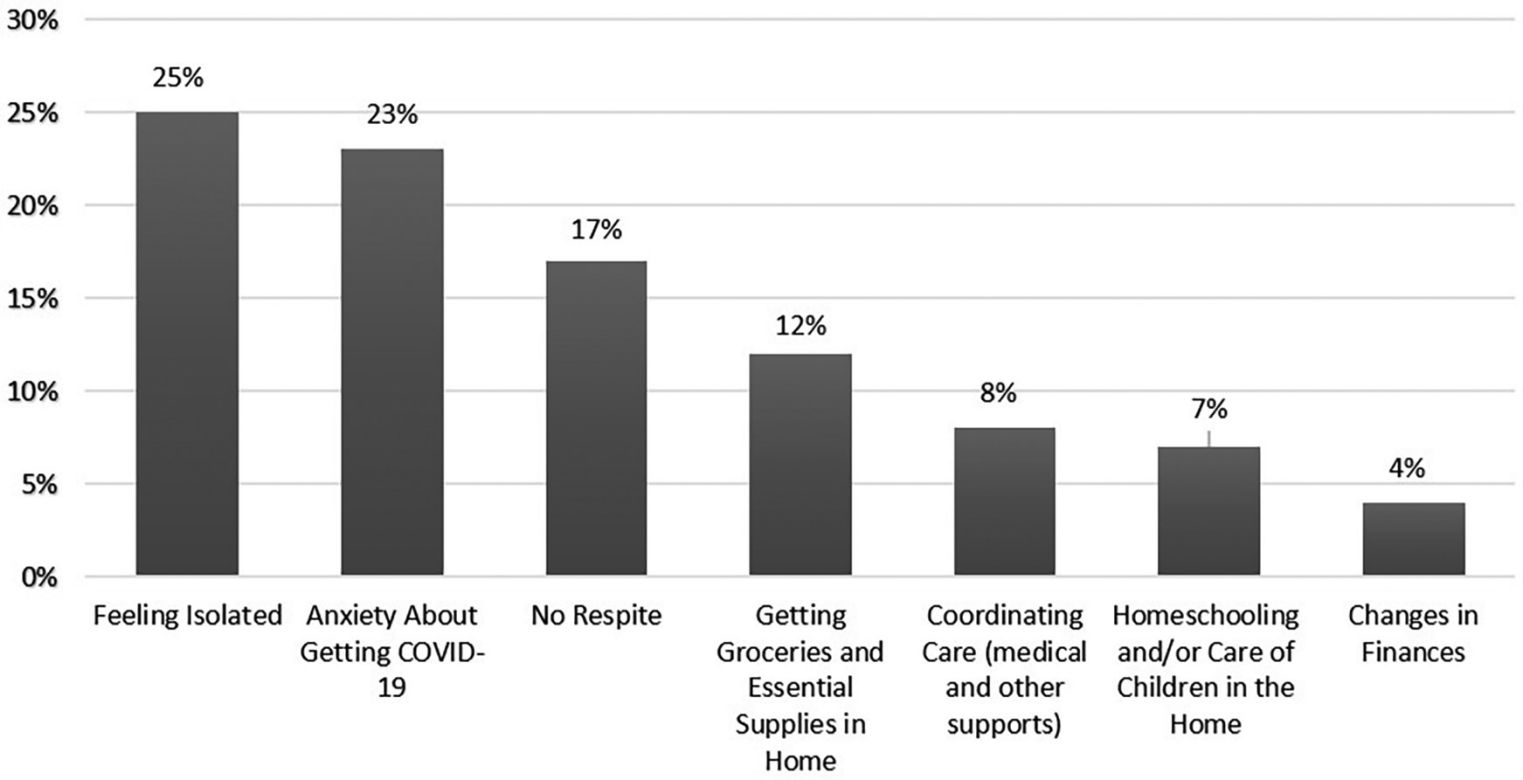
Greatest challenged of being a family caregiver during COVID, as reported by family caregivers during the earliest stages of the COVID-19 pandemic (*N* = 82 family caregivers; spring 2020).

The loss of protected and scheduled respite time was difficult for caregivers, no matter if the caregiver was using a few hours of respite for a weekly grocery trip or whether they had more extensive respite services that allowed for full-time employment. An adult-son caregiver who had increased anxiety without access to his formal respite services during the early-stages of the pandemic reflected that having formal respite gave him “*some time to relax and not worry about, you know, is he going up or down the stairs or is he sneaking out the front door? When you are living with somebody with Alzheimer's, you always got to worry about all these “what ifs”. For those 10 h, I didn't have to worry*”. A woman caring for her aging aunt said, “*It is not what we signed up for. We signed up to help and to take care when we could. Now, it is 24/*7. *I am unable to do my work*”. For these caregivers who were regular users of formal respite, the loss of formal respite felt like a “*breach of contract*” and without the formal services, they had challenges balancing their caregiving, personal, and employment responsibilities. It is, thus, not surprising that “*lack of respite*” was noted by caregivers as one of the greatest stressors associated with the early days of the COVID-19 pandemic. As shown in Figure 1, about 1 in 5 caregivers (17%) said that lack of respite was the greatest challenge they faced during early COVID.

### 3.2 Importance of informal respite

In addition to the loss of formal respite services during COVID, family caregivers also lost the support of other family and friends. In Figure 1, social isolation was noted by about 1 in 4 caregivers as the greatest challenge of COVID, which highlights just how important social connections are to caregivers. Interviews with caregivers highlighted how everyday social connections might serve to provide a source of respite for family caregivers. One caregiver mentioned, “*Seeing friends always gives me a boost. I do not feel so alone*”. Another said, “*even a short hello from a neighbor gives me a moment of pause and normalcy*”. And, finally, one remarked, “*I get by with a little help from my friends*”.

Many caregivers described their caregiving role as “*one of a team of people*” or that they “*tag team each other a lot*”. COVID-19 disrupted the social relationships and tag-teaming that allowed them to share caregiving responsibilities across a care-network:

With COVID, it just shut off, like the lights went out. Nobody came over. Nobody did anything. People think I am a superhero and can handle it. …. What would be helpful is if someone else could come in and give me a break, but no one in the family dares to do that. … They’re too afraid. They’ve quarantined themselves.

Another caregiver commented that family members who used to come over for a weekly dinner were “*so wigged out about getting mom sick, that they just – they essentially drop the food and leave!*” She explains that in the past, someone would often volunteer to take mom to the doctor or out to lunch later that week. However, without the family's Sunday dinners, those conversations were not happening, and she was left as the primary caregiver having to explicitly ask for help, rather than someone casually offering to do something to help. She commented, “*It's caused a lot more tension*”. Another said, “*I no longer had someone available to give me a break*”.

A mother caring for her intellectually disabled adult-child who did not have a large family described a similar feeling of isolation when in-person church services were cancelled. She explained, “*You would see people at least once a week and they would say “How you doing?” …. I've had to say “Hey, I am struggling. Can you just talk to me for a moment?”* … *Now you just have to be more proactive yourself to reach out*”. Although the circumstances of COVID-19 made caregivers become more aware that they needed to be proactive to get help from others, a number commented, “*It is just so hard to ask for help*. *But I now realize how very important it is to ask others to help*. *This is the only way I can get a break*”.

Reflecting upon the industry-wide shut-downs of COVID, one formal care provider feels it necessary to remind families that “*they cannot always count on respite providers*”, while another reflected that “*it's important to view caregiving differently*” and by this, the respite provider meant that caregivers should be “*allowing family, neighbors, or church members to help more*”. Often citing the reality of “*lengthy waitlists for services*” and “*staff shortages*” that have long plagued the direct-care workforce that serves as formal respite providers, respite providers underscore the potential precarity of relying solely on formal respite services and cautioned that formal respite services were unlikely to meet the full needs of caregivers. These insights from both caregivers during COVID and from the providers reflecting on the lessons learned from COVID elevate the importance of informal social support in providing respite to caregivers.

### 3.3 Daily routines as respite

In addition to losing access to both formal and informal respite opportunities, caregivers lamented that their daily routines were lost or disrupted during COVID (e.g., not being able to go to the park for a walk, meet friends for lunch, attend plays/theater, go to grocery store or library, take annual vacations). The loss of these types of routine activities were stressful to caregivers and likely contributed to their feelings of social isolation (refer back to Figure 1); however, this case study also reveals the importance of these daily routines as a possible source of respite for caregivers and care-recipient. Daily routines often provided purposeful, self-affirming, and diversionary social interactions that benefit both caregiver and care recipient.

Prior to COVID, these types of daily routines were not linked to respite, but to the caregivers we interviewed, the loss of them compounded their feelings of social isolation and increased their desire/need for a break. “*It is really painful*”, said one woman when she reflected on the loss of daily routines and special events that she shared with her spouse (for whom she was caring for after a massive stroke) and their children. “*It was the happiest of our happy times when we could get together*”. When daily routines and activities were no longer available, i.e., when so many aspects of our world shutdown during the early stages of the COVID-19 pandemic, this caregiver felt she had absolutely no reprieve or respite from the caregiving role and that she was being denied opportunities for happiness and support from getting together with family and celebrating milestones and celebrations that were not so singularly focused on the tedium of daily caregiving.

The impact of losing daily routines extended beyond the caregivers’ well-being to the care recipient's well-being. Caregivers noted that the loss of routines facilitated feelings of worthlessness and apathy in their care recipient. One individual reflected, “*My mom had outside activities every day that kept her engaged and gave her something to get out of bed for. Now, there is very little to differentiate one day from another*. *This makes it much harder for her to tether short-term memories to anything. –My mom has become frail*”. Many associated the loss of daily routines and support with a decline in their care recipients’ health. One adult daughter caring for her mother noted:

I have little opportunity now to get away from caregiving to socialize, go out to visit anyone … Mom is not able to have other family for company over, so her mood is more unstable. She becomes confused and anxious more often. Her incontinence increases and her sleep quality decreases, so in turn, my rest is interrupted more often. Very stressful for us both.

Both caregivers and providers identified routines that occurred individually, either the caregiver or the care recipient singularly participated in a specific activity, or conjointly, where both the caregiver and care recipient participated in a specific activity. Some caregivers noted that care recipient routines gave the recipient time away from them, the caregiver, which subsequently created a space where both they and their care recipient were able to receive a break or some form of respite from one another. For caregivers reflecting about the challenges of COVID, this loss of mutual respite (as created through daily routines) was felt acutely. In fact, both respite providers and family caregivers began to realize and express how restorative daily routines and activities can be and how these kinds of routine activities may provide a type of break from the everyday responsibilities of caregiving. Thus, the widespread disruptions of daily life that were caused by the COVID pandemic increased awareness that respite should not only be defined as a formal service where caregivers are physically removed from the caregiving environment and relieved from the caregiving role, while the needs of the care recipient are addressed by someone else. Respite might be achieved through daily routines, and sometimes these breaks may be shared with the care-recipient.

### 3.4 Challenges of caregiving without respite

The loss of formal respite services, as well as the disruptions in social relationships and daily routines, increased the contact and proximity between caregivers and care recipients. Many caregivers described a situation where they were with their care recipient “*24/7*”. For caregivers, this constant or persistent contact, coupled with the inability to physically separate from their care recipient, heightened emotional distress. One caregiver who was caring for a spouse with dementia commented,

“I find it more and more difficult to “think for two people”. As a spouse I have tried to create an emotional gap between us, as the person I love/loved disappears a minute at a time. [COVID] made it emotionally more difficult to drive that wedge between us while being couped up 24/7”.

Many caregivers spoke to the challenges that came with increased contact and lack of separation from the care recipient, though some noted benefits of the increased contact that came from not having access to formal and informal respite opportunities. These caregivers framed the increased contact as an opportunity to become more aware of their care recipient. One caregiver reflected how they were able to “*spend more time “watching” behavior*” which subsequently helped them “*understand the disease*” affecting their care recipient which in turn increased their empathy and skill as a caregiver. Although they noted possible benefits, they later remarked that the continued contact “*became more stressful*” over time. Similarly, others commented that the “*unrelenting*” schedule without access to any sort of break or support from others was “*absolutely unsustainable*” for caregivers.

Heightened caregiver stress was not only linked to increased contact or proximity, but also to the increased provision of care. Many caregivers became the sole provider of care, which greatly amplified their burden of care. One caregiver noted that, they “*had the TOTAL weight of responsibility on my shoulders for everything*”. This increased burden of care led to limited or no respite time. Caregivers with limited respite spoke of the increasing necessity for respite; one caregiver who felt particularly isolated and alone commented that respite or “*a break of any kind*” was what they wanted “*more than anything*”. The inability to receive needed or desired respite frequently led to decreased psychological well-being. Many caregivers expressed that their “mental health declined”. Caregivers noted both abrupt and gradual declines in mental health and overwhelming connected their decline to pervading sensations of anxiety, fear, depression, and despondency. For many, this worsening psychological health led to a greater understanding of the importance of respite, no matter how it was provided, formally or informally.

The recognition of respite as necessary led to the increased desire for greater intentionality in respite strategies and use for some caregivers. Caregivers, especially those from the second sample of caregivers who were reflecting on their respite needs during the later (post) stages of COVID, emphasized the need for a respite schedule. One caregiver simply stated, that “*I need a set respite time that I can count on. We all function better when there are things we can count on*”. Many others similarly commented on how they “*learned viscerally*” about the importance and benefit of having “*dependable*” and “*scheduled*” respite time, regardless of who or how the break was achieved.

However, caregivers frequently noted how difficult it was for them to achieve a dependable, regular, or scheduled respite. Reflecting upon the barriers to scheduled respite, one caregiver noted

“I'm sure I'm not alone in that there is no such thing as a regular schedule in our house, so I am not sure at this point that respite involving others would even be feasible because it could be problematic to try to schedule things in advance”.

Additionally, respite service providers identified the many barriers and reasons why caregivers may not always pursue respite, even though they say the need and desire a break. As shown in Table 1, providers commented that caregivers often feel guilt or worry about taking respite or asking others for help, or they lack access financially or practically to use formal respite services. One provider commented that “*these barriers were present well before COVID and still exist today. It is critical to understand them if we are going convince caregivers that they need and deserve a break*”. Another said, “*Respite isn't possible if we cannot figure out how to help caregivers address these barriers*”.

**Table 1:** Barriers and reasons for why caregivers may not use respite, as reported by respite providers.

Barriers/Reason	Mentioned by providers	
	*N*	%
Financial; inability to pay out of pocket for respite services	47	80
Caregiver guilt about taking a break (i.e., leave loved one behind while I get a break)	43	73
Caregiver worry that respite provider won't care for loved one as well as they can	42	71
Lack of available respite workers to hire	42	71
Caregivers don't want to ask or rely on someone else to do “their job” as caregiver	35	59
Waitlists for formal service	28	47
Note sure what respite is	27	46
Too tired or too overwhelmed to even take a break	26	44
Do not know what to do during respite time	21	36
Don't see themselves as a caregiver, so not eligible for a break/respite	20	34

*N* = 59 respite providers in 2023–2024.

Respite providers identified reasons and barriers that potentially interfere or prevent caregivers from using respite. Respite providers often identified multiple barriers.

A common barrier is “*lack of awareness about respite, in general*”. As described in Table 2, providers identified the importance of public awareness and caregiver education, as well as the need to increase the access and availability of respite services. Raising awareness and increasing access were identified as the two most important ways to help caregivers overcome barriers to using respite; and Table 2 provides some specific recommendations suggested by providers. Educating caregivers was viewed by providers as essential for optimizing the benefits of respite care. For one provider, this meant “*Constant education about the importance of respite*” and the importance of emphasizing “*that the care recipient truly benefits from the caregiver getting a much-needed break*”. Others mentioned the importance of educating caregivers about the importance of “*starting respite early, and not just during a crisis*”. One commented that “*Reassurance usually helps greatly*” and that they believe they need to constantly reminding their clients that “*taking breaks gives someone the ability to be the best version of themselves*”. Another said that including this kind of messaging in community-based public awareness campaigns will help *“normalize respite*”, while many other providers emphasized the need to expand access to respite services which would allow more caregivers the opportunity to benefit from respite services.

**Table 2:** Recommendations to help caregivers overcome barriers to using respite.

Increase awareness	Increase resources
• Public awareness campaign, utilizing caregiver testimonials, disseminated via community-based locations (libraries, clinics, schools, grocery stores, media). Recommended messaging includes: ○ importance and benefits of respite to both caregiver and dependent ○ risks and dangers of caregiver burnout ○ Need for early and regular respite, not just during crisis ○ Encouragement of family and friends to provide respite to caregivers in their networks • Trial day of respite • Orientation or support group for new respite users, explicitly addressing their feelings of guilt or worry and outlining how respite will work and how their loved one will be cared for • Educate healthcare and service professionals about respite, so they can provide referrals	• Staff available for consults and care-management to help caregivers learn about respite resources • Drop-in community-based respite centers • Expansion of online caregiver and respite resources, especially to reach rural caregivers • Make respite applications easier and less time consuming; remove need for doctor's note • Professionalize respite provider staff with better pay and education • Increase pool of respite providers (beyond private-pay home care agencies) – perhaps utilize volunteer and peer co-op respite models • Increased funding or voucher programs to “purchase” respite and to reduce out of pocket costs for caregivers

*N* = 59 respite providers in 2023–2024.

### 3.5 Creativity, resilience, and adaptability

This new perspective of respite, in which respite exists beyond the use of formal services, was further demonstrated by how caregivers celebrated their ability to take and benefit from short intentional breaks during the day (See Table 3). Through these breaks and subsequent activities caregivers showed creativity and resilience in the face of the myriad challenges that accompanied the loss of formal services, increased social isolation, and disruption of daily routines that were associated with the pandemic. These types of activities often did not allow the caregiver to fully get-away or even leave the house, yet they still allowed the caregiver an opportunity to “*reset*” or “*distract*” themselves from the stresses of caregiving. Most of these examples are known and recommended methods to practice self-care, though some are focused on occupying or entertaining the care-recipient, as an alternative way for caregivers to take a break that may be meaningful, enjoyable, and diversionary for both care-recipient and caregiver.

**Table 3:** Examples of “short breaks” that family caregivers used in the absence of formal or informal respite services.

Type	Example
Phone	*I can pick up the phone and call a friend and just let it out and talk about it. And it doesn't make it go away, but it helps. It helps balance out the day for myself a little bit. I can do this while still being at home making sure nothing happens to [name].*
Meditation	*Meditation is not part of my thing, but I've been doing it now for about five years. Even if it's ten minutes a day, it's just something that I have to do for me. I think I would be really crazed if I didn't have those ten minutes a day where I quiet my mind.*
Journaling	*I have started journaling … because there are things that happen on a day to day basis that with caregiving that I don't feel is appropriate to share, but that I want to get out. This helps me reset and get back to all the other stuff that has to happen.*
Reading	*I just go in my room and I am just in heaven to just sit quietly and read a book and have nobody bother me*
Cooking	*A lot of my outlets to outside of the house have been cut off, so inside I've been cooking – like everyone else – way more and just trying really stupid, complicated recipes because it distracts me.*
Just 30 min by self	*Last night, I just needed to go on a drive … My kids are in bed. My husband's working. I just drove and got myself a [soda from fast food]. It was so dumb, but it – just having that half-hour to myself, that I was not going to pick something up. I wasn't going to do something for anybody else. I wasn't exercising. I was just doing something that was purely selfish and that, I mean, even that half hour helped.*
Music	*When I'm having a rough day, I crank the tunes and jam out, and find a band that I haven't listened to for a while. Music gets me through everything.*
Service	*I got involved in a service project … I could still have my husband here and everything, but I sewed medical masks. I did over a thousand of those masks. … That was a good opportunity to take my mind away from my own problems to someone else's and being of service to someone else.*
Lessons for care-receiver	*We couldn't really afford it, but I'm telling you, the best money I've spent in the last six months was those guitar lessons for an hour [for her autistic son who did not have access to school/program during COVID]. I get one hour, he's in the backyard with a guitar teacher. He's happy when he comes back. I feel just a bit refreshed too.*

Technology – especially telecommunication and videoconferencing – have become a way for caregivers to get support and may be seen as another creative adaptation to achieve caregiver respite. During the early stages of the pandemic, some caregivers found that family and friends started reaching out more frequently, by phone and video-conferencing. A spousal caregiver whose adult children lived out of town commented, “*We’re actually probably touching bases more, for short intervals. They now call every day to see how their dad is. It is a relief to me to talk to them more frequently. I feel supported and less alone*”. These types of frequent conversations did not necessarily provide caregivers with a physical break, but were seen as helpful and supportive, allowing caregivers to share any changes in the care-recipient's condition, get advice when needed, and find support without having to directly ask for help. The majority of caregivers agreed that they hoped that enhanced communication with the larger family network would continue post-COVID, and hoped that friends and family would continue to reach out to them more often and consistently and “*sometimes*, *just do*” rather than waiting to be asked to help.

As the COVID-19 pandemic persisted and as so many industries had to, a number of formal respite providers used technology to pivot, by replacing their in-person services with online or virtual forms of respite care. These new forms of technology-facilitated respite services were met with mixed reviews. One caregiver was surprised at how convenient and effective virtual respite was for he and her mother: “*She participates two times a day. She likes it. I'm happy to set it up. … It's way more beneficial than what I could do. I am still able to get my work done, while she is entertained*”. Conversely, another explained why virtual respite did not work for them: “*Technology can be so convenient, but for people who have cognitive impairment, it's just, it's hard to relate. … Whenever she is on Zoom, she's like “Is that me? … Oh my Gosh, My Hair! Am I that old?” She's not even involved in the conversation. She is just focused on “What has happened to me?*”

In general, as we are now in a post-pandemic world, caregivers do not view technology-facilitated respite as a viable replacement for in-person respite, which allows for caregivers to have a physical break from caregiving responsibilities; however, many caregivers recognized and conceded the value that technology can and should play in the delivery of respite and caregiver support in the future. In contrast, providers viewed technological services as both an immediate and future alternative to respite care. For some providers, technological adaptations developed in response to the COVID-19 pandemic were integrated into current services and some still persist today. Providers viewed technology as a means to reach more individual caregivers and as a way to reduce some of the barriers to care access. One provider noted, “*We can do a lot to help online and now have some new tools as a result of what we learned during COVID*”. Additionally, technology allowed for future innovation of care. Technology use during COVID was frequently perceived as exploratory, but has been incorporated into continuing practices, such as offering “*virtual options for learning about respite*” and the ability to tailor technological services to make them more “*meaningful and purposeful for individual care recipients*”.

The majority of providers perceived the need to provide innovative care and flexible support as the primary lesson they learned during the pandemic. For one provider, flexibility was contextualized as the “*need to be adaptable, creative, and responsive- especially when there is organizational vulnerability - because the need [for respite] is inelastic*”. Another provider stated that the disruptions to the respite industry caused by the COVID pandemic underscored the importance to “*think outside the box and be flexible*”. Nearly, all respite providers emphasized how much the respite industry had to adapt, and how those adaptations have started to become standard of care in respite industry.

## 4 Discussion

As we reach the five-year anniversary of start of the COVID pandemic, this essay reflects on just how much the COVID-19 pandemic disrupted family caregivers’ access to respite and how both caregivers and respite industry adapted and persisted to these widespread changes over this time. Our data-informed case study, combining data collected from both caregivers and respite providers during the height of COVID and throughout the several years following, reinforce the importance of respite as an essential component of long term supports and services (LTSS) system. This case-study highlights and reconfirms the following five key points about respite:

1) **Respite is among the most desired and needed service or support for caregivers**. Without access to respite, caregivers feel increasing time-burdens and stress associated with the caregiving role. There remains a critical need for continued public awareness of respite and policy initiatives advocating for greater access and expansion to formal respite care services for caregivers.
2) **Families and friends provide meaningful respite to caregivers, through shared care arrangements and others forms of social support directed to the caregiver or care-recipient.** This reinforces the importance of viewing caregiving as shared across care networks, rather than it being assigned solely or exclusively to a single, primary caregiver. This also reinforces the notion that respite can be achieved through informal arrangements, in addition to the delivery of formal services.
3) **Caregivers find joy and diversion from their daily routines.** This finding emphasizes the need for increased caregiver training and education about how to achieve moments of respite through everyday activities and restorative “short breaks” focused on self-care.
4) **Caregivers face barriers, oftentimes self-imposed, that limit their access and willingness to use respite.** This suggests the importance of providing education and support to respite users. Messaging should remind caregivers that they both need and deserve respite and that respite can allow them to be a better caregiver. Supportive messaging may help alleviate the guilt, insecurities, worries, and other feelings that caregivers expressed that self-limit their use of respite.
5) **Technology plays a role in future innovations and delivery of respite and caregiver support** – yet, our results also suggest that technology cannot replace formal respite service or the informal shared arrangements made by caregivers and their network of co-caregivers and supporters that provide true “breaks” from caregiving responsibilities.

In recent years, the U.S. government has adopted policies that recognize the critical role of family caregivers in general (e.g., RAISE Family Caregivers Act, Veterans Administration's Caregiver Support Program, Medicaid home- and community-based waivers to directly compensate family caregivers). Other policies have emphasized the importance of respite as a way to support family caregivers (e.g., Lifespan Respite Care Act, including respite under Medicare hospice benefit) (32). These programs often have stringent eligibility criteria or means-testing, so are not available to all family caregivers. Expansion of these types of state/federal policies would provide greater access and affordability of formal respite care for all family caregivers. This appears to be both needed and desired by family caregivers. Advocacy for expanding access and availability to formal respite services remains priority; respite continues to be among the most needed and desired service for caregivers, and providing respite to caregivers should be considered an essential component of our long term supports and services (LTSS) systems (33).

Our data revealed that caregivers commonly use and benefit from informal “tag-teaming” arrangements made with friends and family, which provides opportunities for respite without reliance on formal respite services. During COVID-19 when so many of our daily routines were disrupted, family caregivers also realized how much they benefitted from their routine daily activities in the community (often shared with care-recipient). Furthermore, caregivers showed creative ways to get intentional short-breaks throughout their day without having to leave the house. Given the importance caregivers placed on these alternative informal moments of respite, the formal definition of respite should be expanded beyond traditional formal respite services (adult day, in-home, residential) to also include the (1) informal arrangements family caregivers have with a care-network that shares caregiving responsibilities and provides routine breaks to the primary caregiver, (2) the everyday routines and activities that emphasize and prioritize the value of and opportunity for caregivers to take regular breaks in and outside of the home. In this regard, **“respite” has been reimagined as an *outcome* that caregivers are seeking, not just a formal service provided by the LTSS system**. This is among the most notable and lasting legacies of the COVID pandemic.

Adopting this expanded definition of respite might be achieved through a public health campaign that highlights the importance of respite, as a way to maintain caregiver's health, well-being, and ability to provide continued care. Based on our data, messaging might highlight themes such as “*offer help, don't wait to be asked*” and “*caregivers need to be cared for, too*”. This kind of messaging should be targeted to all people, not just caregivers, with the goal of emphasizing and recognizing that non-caregivers play a role in supporting caregivers. Additional messaging targeted directly to caregivers may address caregiver hesitancy to use respite; this should emphasize the importance of taking time for self-care, the benefits of planning routine respite time through both formal services or informal arrangements, and the value of taking intentional short breaks, including specific examples of ways to get respite within daily routines and without reliance on formal services. This type of comprehensive public health approach would likely increase opportunities for informal respite, by reducing the public perception that caregiving is provided by a single primary caregiver and by creating public recognition that caregiving should be delivered via partnerships and arrangements that are shared across a network of social and familial relations. These outcomes are ideal because they do not put any additional pressure on the LTSS system, where there is a critical shortage of direct-care workers, limiting the ability of formal respite service industry to meet caregiver needs for a break (34).

That said, it seems shortsighted to recommend only increasing the use of informal respite arrangements, which rely on unpaid exchanges between family members and put even greater expectation on a primary caregivers to arrange their own respite and to maintain relationships within their support networks in order to get respite. The COVID-19 pandemic laid bare the undeniable value that family caregivers bring to the health and well-being of our population, as well as the significant pressures they are under to provide LTSS to our aging, disabled, and chronically ill population with little support from formal health care systems. Thus, the main “call-to-action” arising from these findings is to advocate for state/federal funding and formal policy that would include direct payment, education, and tangible support for family caregivers. This sentiment was aptly shared by a mother to a young child with serious illness and disability:

I have this great fear that after all of this is over that people will have a desire to return to quote-unquote “how things were before” or to a level of normal. I think there are these major issues that’ve been highlighted during COVID … I want to be hopeful that there will be policy change … that there will be change like paid leave, structural caregiver support, and maybe even universal financial support for caregivers, which would be pretty amazing.

We hope that our empirically driven case-study, incorporating data and perspectives of both caregivers and providers from the years spanning early, mid-, and post-pandemic America, provides a reminder of just how critical respite is to caregiver well-being. As well, this case-study provides a set of ideas and innovations for how caregivers might achieve the benefits of respite, even in the face of limited services and long waitlists for those formal services. There remains great interest and need for continued advocacy to increase access and funding for formal respite services and other supportive services and education for caregivers, yet, there also emerged a recognition that “respite” can be achieved through intentional short-breaks (sometimes where the caregiver does not fully detach from the care-recipient or care environment) and through the establishment of shared care arrangements across a care network that build in opportunities for caregivers to achieve respite while someone else is providing care and responsibility to the care-recipient. This re-imagination of “respite” – as an outcome and not just a service to be provided–underlie an important public health mantra that *caregivers need and deserve respite*.

## Data Availability

The datasets presented in this study can be found in online repositories. The names of the repository/repositories and accession number(s) can be found below: DOI: 10.7278/S50d-2rgg-4549 (https://hive.utah.edu/).
